# The Role of Personal Social Networks in Parental Decision-Making for HPV Vaccination: Examining Support and Norms Among Florida Parents

**DOI:** 10.3390/vaccines13070667

**Published:** 2025-06-21

**Authors:** Georges E. Khalil, Carla L. Fisher, Xiaofei Chi, Marta D. Hansen, Gabriela Sanchez, Matthew J. Gurka, Stephanie A. S. Staras

**Affiliations:** 1 Department of Health Outcomes and Biomedical Informatics, University of Florida, Gainesville, FL 32611, USA; georges.khalil.uxr@gmail.com (G.E.K.); carlalfisher@ufl.edu (C.L.F.); m.hansen1@ufl.edu (M.D.H.); gabrielasanchez@usf.edu (G.S.); 2 Department of Pediatrics, University of Florida, Gainesville, FL 32611, USA; xiaofei.chi@ufl.edu; 3 Department of Public Health Sciences, University of Virgina, Charlottesville, VA 22903, USA; mjg3p@virginia.edu

**Keywords:** HPV vaccination, social support, parental intention, social norms, peer influence, public health interventions

## Abstract

**Background**: Human papillomavirus (HPV) vaccination is crucial for preventing HPV-related cancers, yet vaccination rates remain suboptimal, particularly in Florida. Social influence, including family and peer support, may shape parental decisions to vaccinate their children. In this study, we examined the role of social networks (online and offline) in parental intention to vaccinate their 11- to 12-year-old children against HPV. **Methods**: We conducted a cross-sectional survey among 746 parents in Florida as part of the *Text & Talk* trial (2022–2023). Among other questions, parents reported on their intention to vaccinate, perceived social norms, and support received from up to three reported confidants. We performed logistic regression and multivariable analyses to assess the relationship between network support, social norms, and vaccination intent. **Results**: Seventy percent of parents intended to vaccinate their children. Greater support from the first reported confidant was significantly associated with higher vaccination intention (OR = 1.30, *p* < 0.0001). Perceived norms among friends (*p* = 0.01) and higher overall network support (*p* < 0.0001) were also predictive of intent. The higher the percentage of reported family members, the higher the support received for the vaccine (*p* = 0.04). **Conclusions**: Social support, particularly from close confidants and peers, plays a critical role in shaping parental HPV vaccination decisions while accounting for perceived social norms. Public health interventions can leverage peer networks alongside family support to enhance HPV vaccine uptake.

## 1. Introduction

In the United States, there is an urgent need to consider preventive actions for cancer by promoting vaccination against the human papillomavirus (HPV), which may prevent 73% of HPV-related cancers including cervical, oropharyngeal, and vaginal cancers [1]. Particularly, in the state of Florida, adolescent HPV vaccination rates tend to remain below the national average [2]. This is observed for both the initiation of vaccination (1 or more doses) and being up-to-date (UTD; two or three doses depending on age at first dose). Currently, Florida ranks 37th for the initiation rate and 23rd of 50 states for the overall UTD rate [3]. In addition, Florida ranks as the eighth highest among all U.S. states for HPV-associated cancers [4]. To increase cancer prevention with the HPV vaccine, interventions are needed to promote parents’ HPV vaccination uptake for their 11- to 12-year-olds—the recommended age for universal coverage by the US Advisory Committee on Immunization Practices [5].

The Integrated Behavior Model (IBM), a meta-theory combining the Theory of Reasoned Action, the Social Cognitive Theory, and the Theory of Planned Behavior, emphasizes three main factors that drive one’s intention to engage in healthy behavior, including vaccination: attitudes, personal agency, and social norms [6]. The IBM has been evaluated in many HPV vaccine studies and the literature supports that parents’ HPV vaccine acceptance and attitudes are driven by perceptions of disease risk, perceived vaccine effectiveness and safety, personal agency, perceived social norms, and healthcare providers’ recommendations [7,8,9]. Social norms are the shared rules within a group or society that guide acceptable and appropriate behaviors, while the perceptions of social norms are the individual’s belief of what behaviors are expected (injunctive) and what other people are doing (descriptive) [10].

Injunctive and descriptive social norms and perceptions of social norms have been associated with parental HPV vaccination decisions [7,11]; however, their impact is not yet clear when examined alongside interpersonal social support. Research indicates that perceptions of both online and offline social norms play a critical role in HPV vaccine acceptance, with studies showing that perceived norms are associated with parents’ intentions and vaccination for their children [7,12]. In addition to social norms, parents’ interpersonal support can play a key role in their decision to accept vaccination for their children, including the HPV vaccine [13,14,15,16,17,18,19]. A cross-sectional study revealed that mothers with family support were more likely to exhibit positive attitudes toward vaccination [13]. In contrast, social norms can also lead to less protective and even harmful behaviors [20]. For example, having interpersonal contacts advocating against vaccination predicted parents not having their child receive vaccines including the HPV vaccine [14,19]. Building on previous research, further exploration of social support—the emotional, informational, or practical assistance received from one’s interpersonal social network—when accounting for perceived social norms can provide a more comprehensive understanding of the social factors associated with parental HPV vaccination decisions.

In the current study, we aimed to explore relationships between potentially influential social factors in parents’ intent to vaccinate their 11- to 12-year-old Florida-residing child against HPV. First, we examined how parents’ intention to vaccinate is associated with the following: (1) their report of a family member as the first of their most trusted confidants and (2) the percentage of family members among their top three reported confidants and their perceived support for the HPV vaccine. We hypothesized that the HPV vaccine intention is higher among parents with family members as close confidants because close family ties and family dense networks, especially those providing strong support, are associated with positive health outcomes [19,21]. Second, we examined the relationship between their perceived level of support and reported family confidants because prior research suggests that family members are influential for parents’ HPV vaccination decisions [19]. Understanding the relationships between family, support, and parents’ intent to have their child receive the HPV vaccine can help to determine whether interventions should focus on family members and support.

## 2. Materials and Methods

### 2.1. Study Design

All primary caregivers of 11- to 12-year-old patients (e.g., biological parents, adopters, or foster caregivers) were considered eligible for participation in the study. For simplicity, we refer to them as parents in this article. Within the active intervention period of a cluster randomized trial entitled *Text & Talk* conducted between March 2022 and October 2023, 746 parents of 11- to 12-year-olds completed a cross-sectional survey after receiving a vaccine promotion text message (NCT05006833). As part of the study, clinics sent text messages to a random 2/3 of their patients. Text messages informed parents of their child’s eligibility for the HPV vaccine and other adolescent vaccines. Messages were sent at the start of the study for all children who were aged 11 to 12 years old and had not received any of the adolescent vaccines. Between March 2022 and October 2023, parents whose children turned 11 years old were sent a message approximately 2 weeks prior to their birthday. All parents who were sent a text message were sent an invitation to complete a brief behavioral survey.

### 2.2. Survey Procedures

Two to three days after the vaccine reminder messages, parents were sent an additional text message inviting them to take a one-time, cross-sectional survey evaluating parents’ acceptance of text messages and the IBM targeted constructs using questions adopted or adapted from prior studies [6,22,23,24,25]. The included survey questions are provided in the Supplementary Materials. Survey invitations included a personal link to the REDCap survey. Parents who did not complete the survey and did not respond STOP to the survey invitation text message were sent up to two reminders via text message. Parents who did not respond to text messages were contacted up to three times by phone to remind them of the survey and give them an opportunity to complete the survey over the phone. Parents who chose to complete the survey, sent via text message, signed a consent form in REDCap. When the parent chose to complete the survey over the phone, study staff explained the participant’s rights when participating in research and accepted verbal agreement under a waiver of documentation of informed consent. Regardless of survey mode, parents were sent USD 30 for survey completion. All study procedures were approved by the University of Florida Institutional Review Board.

### 2.3. Main Measures

The main outcome of our study was parents’ intention to have their child vaccinated for HPV. Intention to vaccinate their child was measured by asking respondents: “How likely is it that your child will receive the HPV shots in the next 12 months?”. This question was derived from the National Immunization Survey and had five response options: very likely, somewhat likely, not too likely, not at all likely, and don’t know [23]. For analysis, similar to a prior study [26], we dichotomized the intention into intend to vaccinate (very likely and somewhat likely) and do not intend to vaccinate (not too likely, not at all likely, and don’t know).

The considered predictors were social norms measured with 9 items adapted or adopted from prior studies. First, we measured parents’ perceived norms of family and close friends (one item each). Adapted from prior studies [27,28], parents were asked their agreement with the following statement: “My family/close friends would want me to get the HPV vaccine for my child.” The response options were on a five-item Likert scale from not at all to definitely yes. For general perceived norms to vaccinate their children, two questions were asked: one for daughters and one for sons. Based on a prior study of substance-use norms [29], participants were asked to think about a group of 100 parents who have a daughter/son of their child’s age and answer how many of these parents they think have gotten the HPV vaccine for their daughter/son. The response options were 10 intervals of 10 ranging from 0 to 100.

To determine parents’ top social connections, we asked participants to identify up to three individuals from their social network, who they would talk to about important confidential matters [30,31]. For each reported person, participants were asked to answer four questions pertaining to each reported social contact. The first question asked parents to indicate how they knew this person. Answer choices were “family member”, “friend”, “neighbor”, “work colleague”, “other, specify”, and “I talk to no one about confidential matters”. The second question asked parents about the level of general support (GS) which was adapted from commonly used measures for perceived support and acceptance within social networks: “How supportive of your decisions is this person?” [32,33]. Response options on a 4-point scale ranged from “extremely supportive” to “not at all supportive”. The third question was adapted from previous research to measure the level of influence (IN): “How influential is this person in your life”? [34,35]. Response options on a 4-point scale ranged from “extremely influential” to “not at all influential”. The fourth question measured the level of the connection’s support of the HPV vaccine (R): “How likely is this person to suggest the HPV vaccine for your child?”. The answer choices ranged from “very likely” to “not at all likely” on a 4-point scale. This question was a novel measure intended to reflect the importance of family members’ approval of the HPV vaccine on parents’ intention to have their child receive the vaccine [36,37].

For analysis, we combined the items above to create constructs. First, we measured the concept of cohesion exposure, which is the percentage of reported contacts who were family members [25]. To do so, we calculated the number of reported family members divided by the total number of reported individuals for each participant [38]. We measured support for HPV vaccination from the first reported person (S_1_) in two steps. First, we averaged GS and IN from the first reported person. This average was then multiplied by R from the first reported person. This measure was continuous and ranged from 0 to 16. The support for HPV vaccination from all reported individuals (S) was measured by adding up the support for HPV vaccination from each reported person. This measure was continuous and ranged from 0 to 48. As a result, we were able to combine two concepts: (1) how much participants’ connections generally influence their decisions and (2) how often their connections recommend the HPV vaccine.

### 2.4. Data Analysis

Data were summarized using percentages and frequencies for all categorical and ordinal variables of interest. We also examined the relationship between participant characteristics and vaccination intent to identify potential covariates that can be included in the models. Chi-square tests were conducted for categorical variables, and Cochran–Armitage trend tests were used for ordinal variables to compare demographic differences between parents who intended to have their child vaccinated and those who did not. As a result, the marital status and the number of 11- to 12-year-old children were included as covariates in the subsequent models. Race/ethnicity was also included based on previous epidemiological evidence [39]. We used logistic regression to assess the odds of parents intending to vaccinate their child. Additionally, multivariable linear regression analysis was performed to identify significant predictors related to receiving support from a family member as the first confidant and support from a number of reported family members, respectively. The same demographic variables were included as covariates in all models. All analyses were conducted using Statistical Analysis Software (SAS) Version 9.4 (SAS Institute Inc., Cary, NC, USA) , with statistical significance set at *p* < 0.05.

## 3. Results

### 3.1. Participant Characteristics

The study covered 746 parents residing in North Central Florida, 681 were female (91%) and 59 were male (8%) (Table 1). The parents represented a diversity of racial/ethnic groups with 42% non-Hispanic White, 30% Hispanic, and 21% non-Hispanic Black. Of our participants, 47.2% reported English as their language of choice. Based on the American Association of Public Opinion research response rate 4 [40], we achieved a 21% response rate with a 59% cooperation rate.

### 3.2. Intention to Vaccinate Their Child Against HPV

Among participating parents, 519 (70%) reported that they intended to vaccinate their children against HPV within the next 12 months. In bivariate analyses, having two or more children (*p* < 0.01), having at least one boy (*p* < 0.05), and being married or living with a significant other (*p* < 0.05) were related to an intention to vaccinate.

### 3.3. Nominations of Confidants

Among the 746 parents, 678 (91%) parents reported at least one person they talk to about important confidential matters. Based on their nominations, 511 (69%) reported a family member as the first person they would talk to about important confidential matters. Among parents that reported at least one person, 624 (82%) reported at least one family member.

Participating parents scored an average of 10.54 (SD = 4.20) out of 16 in support for HPV vaccination from the first reported person. They scored an average of 29.49 (SD = 11.99) out of 48 in support for HPV vaccination from all reported confidants.

### 3.4. HPV Vaccination Intent and First Reported Confidant

We examined the association between HPV vaccine intent and selection of a family member as the first confidant and the parent’s perception of the level of support they receive from this person (Table 2). Controlling for the confidant’s relationship, a higher perceived level of support received from the first reported confidant was significantly associated with a higher intention to vaccinate (OR = 1.30, *p* < 0.0001). Above and beyond associations with the confidant’s support and relationship, intention to vaccinate was also associated with a perceived norm for the HPV vaccine among friends (“definitely yes” versus “not at all”: OR = 1.78, *p* = 0.01) and having two or more children (0R = 1.87, *p* = 0.03).

With our second model, we examined the percentage of reported family members and the perceived level support from all reported confidants for the HPV vaccine specifically (Table 3). The perceived level of support for the HPV vaccine from all reported confidants was associated with a higher likelihood of intention to vaccinate (OR = 1.10, *p* < 0.0001). Above and beyond associations with the confidants’ support and relationships, parents’ vaccination intent was associated with the perceived norms from family and friends, as well as parents’ marital status.

### 3.5. Association Between Perceived Support for the HPV Vaccine and Reporting Family Members

To investigate whether family members as the first confidants were associated with the likelihood of perceived support for the HPV vaccine, we examined the association between reporting the first confidant to be a family member and the level of support for the HPV vaccine from the first reported confidant (Table 4). The perceived level of support for the HPV vaccine from the first confidant was higher when the first confidant was a family member compared to a non-family member (*p* < 0.0001). Beyond the effects of family relation, a higher level of perceived support for the HPV vaccine from the first reported confidant was associated with perceived norms from family members (*p* < 0.0001), perceived norms to vaccinate a daughter (*p* < 0.001), being Hispanic compared to non-Hispanic White (*p* = 0.01), and marital status.

With our last model, we examined the association between the perceived level of support for the HPV vaccine from all reported confidants and the percentage of reported family members (Table 5). The perceived support of confidants for the HPV vaccine was higher with a higher percentage of reported family members (*p* = 0.04). Beyond the effects of confidants’ relationships, increased perceived support for the HPV vaccine from reported confidants was associated with perceived norms from family members (definitely yes vs. not at all *p* = 0.04), perceived norms to vaccinate a daughter (0–19 versus 40–59, *p* = 0.01, and 0–19 versus 80–100, *p* = 0.002), and being non-Hispanic White compared Hispanic (*p* = 0.0002) or non-Hispanic Black (*p* = 0.01).

## 4. Discussion

The findings from our study underscore the significant role that social support plays in parents’ intention to vaccinate their children against HPV. Specifically, the support received from the first reported confidant was strongly associated with a higher intention to vaccinate. This result aligns with the existing literature that emphasizes the importance of close interpersonal relationships in health-related decision-making [41]. For instance, studies have shown that support from family and friends can increase the likelihood of getting vaccinated through encouragement or modeling behavior [42,43]. In addition, observational research indicates that vaccinated individuals often perceive stronger social influence from their immediate social circles than those who are unvaccinated [44]. Our results further reveal that receiving support from all three reported confidants was significantly related to a higher likelihood of intention to vaccinate. This finding is consistent with the theory of social capital, which states that cumulative support from a network can strengthen individual health behaviors [45].

While the current study indicates that reporting support from confidants is important, it did not demonstrate that reporting family members as confidants directly translates into a higher likelihood of vaccination intention, given it was not significantly associated with a higher intention to vaccinate. While having family members as confidants is generally an influential social factor in health behavior decisions [46], in the context of the HPV vaccination of 11- to 12-year-olds, parents’ peer support may be especially important to consider in identifying new intervention strategies. While family members are important, public health interventions aiming to increase HPV vaccination rates specifically should consider broadening their focus beyond the family unit to include other influential figures within a parent’s social network, particularly friends and healthcare providers.

Another key finding from the current study is the association between perceived norms and vaccination intentions. The perceived norms among friends were significantly associated with a higher intention to vaccinate, while the perceived norms from family members were not as influential. This result supports the previous literature on the importance of social norms within peer groups in shaping health behaviors and HPV vaccination intentions [10]. However, the finding that family norms were less influential is intriguing and may suggest that parents can be more swayed by the opinions and behaviors of non-familial ties than by their family when it comes to making vaccination decisions for their children. Family involves multi-generational relationships with variant sociohistorical experiences as well as different developmental tasks [11,47], whereas parents’ chosen (and prioritized) friendships likely reflect more similarities both in generation and social experiences [19]. Thus, these parents may find the advice and support of other parents most important in their HPV vaccination uptake [19]. This is usually the case for younger populations who find their friends to be a more relevant and immediate source of social comparison than family members [48]. Nearly half of the participants in this study are young adults between 35 and 44 years of age, who may find friends to be crucial influencers of their decisions. It is also possible, as this is a cross-sectional study, that parents are likely to choose friends who have similar vaccine attitudes as themselves while they do not have the freedom to choose their family members. Future research could explore how these norms are formed and disseminated within different social groups, providing further insight into how to harness them to improve vaccination rates, potentially by tailoring interventions to peer networks and emphasizing the positive behaviors of others in the community.

Despite the lesser influence of family members on vaccination intentions, our study found that participants reported a higher level of support from their first confidant if this individual was a family member. In addition, reporting a higher percentage of family members was associated with a higher level of support. These results highlight an intricate psychosocial dynamic. While family members may not directly influence vaccination intentions, they may still provide substantial emotional and instrumental support. The findings agree with the broader research on social support, which tends to differentiate between the emotional comfort provided by family and the informational or practical support that might come from other network ties [49,50]. This suggests that family members can provide a supportive environment that is more complementary than direct when influencing health behaviors like vaccination. Future research could delve into the different types of support received from family versus non-family members and how they impact health decision-making processes.

Several study limitations must be noted. First, as we used a cross-sectional design, we cannot establish causality nor directionality between variables. Therefore, it is possible that intentions influence how parents perceive their friends and family. Second, while we measured parents’ intention to vaccinate, a highly predictive factor for vaccine receipt [18], we did not follow parents to determine if intention led to vaccination. Therefore, it is possible that the associations between perceptions of social support and vaccination are different than those with the intention to vaccinate. Third, the measurement of social support in this study was based on participants’ subjective perceptions. While perceptions of social support are consistently linked to health outcomes [51], individuals tend to overestimate their peers’ agreement with their attitudes in general and about vaccines [52,53]. Thus, if we collected friend and family members’ actual attitudes about vaccines, we may reach different conclusions. While parents’ perception of peer attitudes may be more influential on their behaviors than their peers’ actual attitudes [54], including members of parents’ social networks in the study would allow for a more comprehensive measurement of actual social support. Through this approach, researchers can analyze the social network structures as an indicator of social support, offering deeper insights into how these networks influence parental decisions.

Nevertheless, this study presents some notable strengths. We successfully recruited a racially and ethnically diverse sample of primary caregivers with 42% non-Hispanic White, 21% non-Hispanic Black, and 30% Hispanic participants, thereby contributing to the generalizability of the findings across racial/ethnic groups and very similar to the racial/ethnic distribution of middle-aged adults in Florida [55]. For example, among 35- to 39-year-olds, 45% are non-Hispanic White, 17% are non-Hispanic Black, and 32% are Hispanic [55]. Our sample of primarily females is consistent with the predominance of women as primary caregivers of children in the United States and worldwide [56]. Second, this study allowed survey assessments to take place through a diverse number of media channels, including phone and online surveys. This strategy allows parents to engage based on their comfort. Additionally, this study was the first to strategically measure the general influence of social networks and their support for the HPV vaccine separately before integrating these factors under one measure. This approach mitigated the risk of survey respondents downplaying the role that social influence has on their thoughts and decisions about the vaccine.

## 5. Conclusions

Ultimately, this study offers important contributions to our understanding of the social factors influencing HPV vaccination decisions. The findings suggest while general support from parents’ networks can play a key role in vaccination intentions, the presence of family members in one’s network of confidants is associated with a higher level of support. These results emphasize the importance of incorporating peer and familial support dynamics into public health interventions. Practitioners in this domain should consider design strategies that engage peer networks, community groups, and particularly family members. Future research should also continue to explore the nuances of social support and its impact on vaccination, particularly in diverse populations. While this study focused on interpersonal and family relationships, other influences in cultural and society, particularly the media and social media, influence social norms and parents’ vaccine decisions [57,58]. It may be useful to explore how social media plays a role in shaping social norms within familial or relational networks as it has also been demonstrated to be an effective influential factor in shaping families’ health beliefs and related behavioral intentions [59]. Moreover, investigating the mechanisms by which social support influences decision-making could yield insights that further refine public health messaging and intervention strategies.

## Figures and Tables

**Table 1:** Participant characteristics and intention to have their child receive the HPV vaccine.

	All		Not Intending to Vaccinate		Intending to Vaccinate		*p*-Value *
	N	% of Total	N	% of Given Characteristic	N	% of Given Characteristic	
**Total**	746	100%	227	30%	519	70%	
**Gender**	5	1%	3	60%	2	40%	0.25
Missing							
Male	59	8%	20	34%	39	66%	
Female	681	91%	203	30%	478	70%	
Other	1	0%	1	100%	0	0%	
**Race/Ethnicity**	225	30%	65	29%	160	71%	0.56
Hispanic							
Non-Hispanic White	313	42%	101	32%	212	68%	
Non-Hispanic Black	160	21%	50	31%	110	69%	
Other	48	6%	11	23%	37	77%	
**Age (years)**							0.47
Missing	18	2%	5	28%	13	72%	
25–34	186	25%	51	27%	135	73%	
35–44	362	49%	111	31%	251	69%	
45–72	180	24%	60	33%	120	67%	
**Number of 11- to 12-year-old children**							**0.01**
Missing	21	3%	15	71%	6	29%	
1	556	74%	176	32%	380	68%	
2 or more	169	23%	36	21%	133	79%	
**Number of 11- to 12-year-old girls they have**							0.33
Missing	61	8%	25	41%	36	59%	
0	272	37%	81	30%	191	70%	
1	365	49%	113	31%	252	69%	
2 or more	48	6%	8	17%	40	83%	
**Number of 11- to 12-year-old boys they have**							**0.01**
Missing	69	9%	23	33%	46	67%	
0	247	33%	88	36%	159	64%	
1	389	52%	107	28%	282	72%	
2 or more	41	6%	9	22%	32	78%	
**Education level**							0.09
Missing	5	1%	3	60%	2	40%	
High school or less	154	21%	40	26%	114	74%	
Some post high school education	313	42%	87	28%	226	72%	
College degree	163	22%	61	37%	102	63%	
Postgraduate degree	111	15%	36	32%	75	68%	
**Marital status**							**0.03**
Missing	6	1%	4	67%	2	33%	
Never married	122	16%	33	27%	89	73%	
Married or not married, but living together	479	64%	159	33%	320	67%	
Separated, divorced, or widowed	139	19%	31	25%	108	40%	

* *p*-value from Cochran–Armitage trend test, otherwise from Chi-square test. All statistically significant *p*-values are in bold font.

**Table 2:** Adjusted association of intention to vaccinate with first confidant relationship and support.

Intent to Vaccinate		N or Mean	% or SD	OR	95% Wald CI		*p*-Value
**Reporting** **the first confidant to be a family member**	Yes	477	75.8%	0.69	0.41	1.16	0.16
	No (ref. *)	152	24.2%	-	-	-	
							
**Perceived level of support from the first reported confidant**		10.6	4.2	1.30	1.22	1.39	**<0.0001**
**Perceived norms from family**							
	Definitely yes	173	27.5%	1.34	0.37	4.89	0.21
*“My family would want me to get the HPV vaccine for my child”*	Probably yes	141	22.4%	0.74	0.23	2.36	
	Possibly	192	30.5%	0.49	0.17	1.37	
	Probably not	72	11.5%	0.55	0.19	1.57	
	Not at all (ref.)	51	8.1%	-	-	-	
**Perceived norms from friends**							
	Definitely yes	128	20.3%	1.78	0.43	7.37	**0.01**
*“My close friends would want me to get the HPV vaccine for my child”*	Probably yes	162	25.8%	5.70	1.44	22.55	
	Possibly	221	35.1%	2.43	0.72	8.17	
	Probably not	81	12.9%	0.86	0.26	2.90	
	Not at all (ref.)	37	5.9%	-	-	-	
**Perceived norms to vaccinate a son**							
*“Think about a group of 100 parents who have a son of your child’s age. How many of them do you think have gotten the HPV vaccine for their son?”*	0–19 (ref.)	216	34.3%	-	-	-	0.51
	20–39	127	20.2%	1.44	0.66	3.11	
	40–59	142	22.6%	1.82	0.79	4.21	
	60–79	81	12.9%	1.80	0.62	5.22	
	80–100	63	10.0%	0.80	0.21	3.09	
**Perceived norms to vaccinate a daughter**							
*“Think about a group of 100 parents who have a daughter of your child’s age. How many of them do you think have gotten the HPV vaccine for their daughter?”*	0–19 (ref.)	145	23.0%	-	-	-	0.20
	20–39	91	14.5%	1.33	0.58	3.08	
	40–59	167	26.6%	0.98	0.42	2.30	
	60–79	114	18.1%	2.19	0.81	5.90	
	80–100	112	17.8%	2.62	0.79	8.70	
**Race/Ethnicity**							
	Hispanic	176	28.0%	1.05	0.60	1.82	0.07
	Non-Hispanic Black	136	21.6%	1.30	0.70	2.41	
	Other	40	6.4%	1.64	0.58	4.62	
	Non-Hispanic White (ref.)	277	44.0%	-	-	-	
**Marital Status**							
	Married or not married, but living together	408	64.8%	0.75	0.40	1.40	0.13
	Separated, divorced, or widowed	118	18.8%	1.40	0.63	3.11	
	Never married (ref.)	103	16.4%	-	-	-	
**Number of 11–12 y.o. children**							
	2 or more	145	23.0%	1.87	1.05	3.32	**0.03**
	One child (ref.)	484	77.0%	-	-	-	

* ref. stands for the reference factor.All statistically significant p-values are in bold font.

**Table 3:** Adjusted association of intention to vaccinate with the percentage of reported family members and support for the HPV vaccine from all reported confidants.

Intent to Vaccinate		N or Mean	% or SD	OR	95% Wald CI		*p*-Value
**Percentage** **of reported family members**		64.9%	33.5%	1.00	0.99	1.00	0.47
**Level of support for the HPV vaccine from all reported individuals**		24.9	12.0	1.10	1.07	1.12	**<0.0001**
**Perceived norms from family**							
	Definitely yes	174	27.5%	2.32	0.66	8.10	0.05
*“My family would want me to get the HPV vaccine for my child”*	Probably yes	142	22.5%	0.99	0.31	3.13	
	Possibly	192	30.4%	0.61	0.22	1.69	
	Probably not	73	11.6%	0.62	0.22	1.74	
	Not at all (ref. *)	51	8.0%	-	-	-	
**Perceived norms from friends**							
	Definitely yes	129	20.4%	1.63	0.42	6.25	**0.001**
*“My close friends would want me to get the HPV vaccine for my child”*	Probably yes	162	25.6%	5.81	1.56	21.62	
	Possibly	223	35.3%	2.48	0.79	7.84	
	Probably not	81	12.8%	0.74	0.23	2.35	
	Not at all (ref. *)	37	5.9%	-	-	-	
**Perceived norms to vaccinate a son**							
*“Think about a group of 100 parents who have a son of your child’s age. How many of them do you think have gotten the HPV vaccine for their son”?*	0–19 (ref.)	216	34.2%	-	-	-	0.70
	20–39	129	20.4%	1.18	0.55	2.56	
	40–59	142	22.5%	1.53	0.66	3.57	
	60–79	81	12.8%	1.79	0.62	5.19	
	80–100	64	10.1%	0.87	0.23	3.26	
**Perceived norms to vaccinate a daughter**							
*“Think about a group of 100 parents who have a daughter of your child’s age. How many of them do you think have gotten the HPV vaccine for their daughter”?*	0–19 (ref.)	145	22.9%	-	-	-	0.06
	20–39	92	14.6%	1.86	0.81	4.24	
	40–59	167	26.4%	1.35	0.58	3.14	
	60–79	115	18.2%	3.17	1.18	8.46	
	80–100	113	17.9%	3.69	1.16	11.81	
**Ethnicity**							
	Hispanic	177	28.0%	1.25	0.72	2.17	0.45
	Non-Hispanic Black	137	21.7%	1.57	0.85	2.91	
	Other	41	6.5%	1.65	0.60	4.57	
	Non-Hispanic White (ref.)	277	43.8%	-	-	-	
**Marital status**							
	Married or not married, but living together	411	65.0%	0.62	0.33	1.15	**0.03**
	Separated, divorced, or widowed	118	18.7%	1.35	0.61	2.99	

* ref. stands for the reference factor.All statistically significant *p*-values are in bold font.

**Table 4:** Adjusted association of the perceived level of support for the HPV vaccine from the first reported confidant with first confidant’s relationship.

Predictors of Level of Support		N	%	Beta	SE	*p*-Value
**Reporting** **the first confidant to be a family member**	Yes	477	24.2%	1.57	0.34	**<0.0001**
	No (ref. *)	152	75.8%	-	-	
**Perceived norms from family**						
*“My family would want me to get the HPV vaccine for my child”*	Definitely yes	173	27.5%	3.37	0.81	**<0.0001**
	Probably yes	141	22.4%	1.08	0.81	0.18
	Possibly	192	30.5%	0.72	0.75	0.34
	Probably not	72	11.5%	−0.45	0.76	0.56
	Not at all (ref)	51	8.1%	-	-	
**Perceived norms from friends**						
*“My close friends would want me to get the HPV vaccine for my child”*	Definitely yes	128	20.3%	0.47	0.92	0.61
	Probably yes	162	25.8%	−0.45	0.90	0.61
	Possibly	221	35.1%	−1.02	0.84	0.22
	Probably not	81	12.9%	−1.67	0.83	0.05
	Not at all (ref)	37	5.9%	-	-	
**Perceived norms to vaccinate a son**						
*“Think about a group of 100 parents who have a son of your child’s age. How many of them do you think have gotten the HPV vaccine for their son”?*	0–19 (ref)	216	34.3%	-	-	
	20–39	127	20.2%	−0.75	0.49	0.13
	40–59	142	22.6%	−0.25	0.52	0.64
	60–79	81	12.9%	−0.32	0.61	0.60
	80–100	63	10.0%	0.02	0.72	0.98
**Perceived norms to vaccinate a daughter**						
*“Think about a group of 100 parents who have a daughter of your child’s age. How many of them do you think have gotten the HPV vaccine for their daughter”?*	0–19 (ref)	145	23.1%	-	-	
	20–39	91	14.5%	1.76	0.56	**0.002**
	40–59	167	26.6%	2.14	0.55	**0.0001**
	60–79	114	18.1%	2.15	0.60	**0.0004**
	80–100	112	17.8%	3.01	0.67	**<0.0001**
**Race/Ethnicity**						
	Hispanic	176	28.0%	−0.91	0.35	**0.01**
	Non-Hispanic Black	136	21.6%	−0.33	0.39	0.40
	Other	40	6.4%	−0.21	0.60	0.72
	Non-Hispanic White (ref)	277	44.0%	-	-	
**Marital status**						
	Married or not married, but living together	408	64.8%	−0.97	0.41	**0.02**
	Separated, divorced, or widowed	118	18.8%	−1.01	0.49	**0.04**
	Never married (ref)	103	16.4%	-	-	
**Number of 11–12 y.o. children**						
	2 or more	145	23.1%	0.19	0.34	0.57
	One child (ref)	484	76.9%	-	-	

* ref. stands for the reference factor. All statistically significant *p*-values are in bold font.

**Table 5:** Adjusted association of the perceived level of support for the HPV vaccine from all reported confidants with the percentage of reported family members.

Predictors of Level of Support		N or Mean	% or SD	Beta	SE	*p*-Value
**Percentage** **of reported family members**		64.9%	33.5%	0.03	0.01	**0.04**
**Perceived norms from family**						
	Definitely yes	174	27.5%	5.21	2.49	**0.04**
*“My family would want me to get the HPV vaccine for my child”*	Probably yes	142	22.5%	−1.69	2.47	0.49
	Possibly	192	30.4%	−1.27	2.28	0.58
	Probably not	73	11.5%	−3.14	2.34	0.18
	Not at all (ref. *)	51	8.1%	-	-	
**Perceived norms from friends**						
	Definitely yes	129	20.4%	2.91	2.81	0.30
*“My close friends would want me to get the HPV vaccine for my child”*	Probably yes	162	25.6%	−0.40	2.74	0.89
	Possibly	223	35.3%	−2.41	2.56	0.35
	Probably not	81	12.8%	−2.69	2.56	0.29
	Not at all (ref)	37	5.9%	-	-	
**Perceived norms to vaccinate a son**						
*“Think about a group of 100 parents who have a son of your child’s age. How many of them do you think have gotten the HPV vaccine for their son”?*	0–19 (ref)	216	34.2%	-	-	
	20–39	129	20.4%	−0.36	1.50	0.81
	40–59	142	22.5%	−0.41	1.60	0.80
	60–79	81	12.8%	−0.54	1.86	0.77
	80–100	64	10.1%	−0.50	2.19	0.82
**Perceived norms to vaccinate a daughter**						
*“Think about a group of 100 parents who have a daughter of your child’s age. How many of them do you think have gotten the HPV vaccine for their daughter”?*	0–19 (ref)	145	22.9%	-	-	
	20–39	92	14.6%	1.86	1.72	0.28
	40–59	167	26.4%	4.49	1.69	**0.01**
	60–79	115	18.2%	2.94	1.83	0.11
	80–100	113	17.9%	6.38	2.03	**0.002**
**Race/Ethnicity**						
	Hispanic	177	28.0%	−4.04	1.07	**0.0002**
	Non-Hispanic Black	137	21.7%	−3.02	1.19	**0.01**
	Other	41	6.5%	0.09	1.81	0.96
	Non-Hispanic White (ref)	277	43.8%	-	-	
**Marital status**						
	Married or not married, but living together	411	65.0%	−1.75	1.25	0.16
	Separated, divorced, or widowed	118	18.7%	−2.45	1.49	0.10
	Never married (ref)	103	16.3%	-	-	
**Number of 11–12 y.o. children**						
	2 or more	147	23.3%	1.91	1.04	0.07
	One child (ref)	485	76.7%	-	-	

* ref. stands for the reference factor.All statistically significant p-values are in bold font.

## Data Availability

Data presented in the study were not approved by IRB for open access. Requests can be made to the corresponding author to seek approval for sharing data.

## Supplementary Materials

The following supporting information can be downloaded at: https://www.mdpi.com/article/10.3390/vaccines13070667/s1, Survey questions are provided verbatim in the order they appear in the manuscript tables.

## References

[1] Senkomago V. (2019). Human Papillomavirus–Attributable Cancers—United States, 2012–2016. Morb. Mortal. Wkly. Rep..

[2] Pingali C., Yankey D., Chen M. (2024). National Vaccination Coverage Among Adolescents Aged 13–17 Years—National Immunization Survey-Teen, United States, 2023. Morb. Mortal. Wkly. Rep..

[3] Centers for Disease Control and Prevention (CDC) TeenVaxView. https://www.cdc.gov/teenvaxview/interactive/index.html.

[4] U.S. Department of Health and Human Services, Centers for Disease Control and Prevention (CDC), National Cancer Institute (NCI) Cancer Statistics Working Group. U.S. Cancer Statistics Data Visualizations Tool. https://gis.cdc.gov/cancer/USCS/#/AtAGlance/.

[5] Meites E., Szilagyi P.G., Chesson H.W., Unger E.R., Romero J.R., Markowitz L.E. (2019). Human Papillomavirus Vaccination for Adults: Updated Recommendations of the Advisory Committee on Immunization Practices. Morb. Mortal. Wkly. Rep..

[6] Montaño D.E., Kasprzyk D. (2015). Theory of reasoned action, theory of planned behavior, and the integrated behavioral model. Health Behavior: Theory, Research, and Practice.

[7] Balogun F.M., Omotade O.O. (2022). Parental intention to vaccinate adolescents with HPV vaccine in selected communities in Ibadan, Southwest Nigeria: An application of Integrated Behavioral Model. Hum. Vaccines Immunother..

[8] Degarege A., Krupp K., Fennie K., Srinivas V., Li T., Stephens D.P., Madhivanan P. (2019). An integrative behavior theory derived model to assess factors affecting HPV vaccine acceptance using structural equation modeling. Vaccine.

[9] Rodriguez S.A., Mullen P.D., Lopez D.M., Savas L.S., Fernández M.E. (2020). Factors associated with adolescent HPV vaccination in the U.S.: A systematic review of reviews and multilevel framework to inform intervention development. Prev. Med..

[10] Smith J.R., Louis W.R. (2008). Do as we say and as we do: The interplay of descriptive and injunctive group norms in the attitude-behaviour relationship. Br. J. Soc. Psychol..

[11] Wang Y., Chen Y., Bao S. (2023). The impact of exposure to HPV related information and injunctive norms on young women’s intentions to receive the HPV vaccine in China: A structural equation model based on KAP theory. Front. Public Health.

[12] Stout M.E., Christy S.M., Winger J.G., Vadaparampil S.T., Mosher C.E. (2020). Self-efficacy and HPV Vaccine Attitudes Mediate the Relationship Between Social Norms and Intentions to Receive the HPV Vaccine Among College Students. J. Community Health.

[13] Handinis S., Mochammad B.Q., Dominicus H. (2018). The Effect of Family Support and Immunization Services Access on Mothers Attitudes in Providing Basic Immunization. Int. J. Public Health Clin. Sci..

[14] Brunson E.K. (2013). The impact of social networks on parents’ vaccination decisions. Pediatrics.

[15] Kahn J.A., Rosenthal S.L., Jin Y., Huang B., Namakydoust A., Zimet G.D. (2008). Rates of human papillomavirus vaccination, attitudes about vaccination, and human papillomavirus prevalence in young women. Obstet. Gynecol..

[16] Dempsey A.F., Zimet G.D., Davis R.L., Koutsky L. (2006). Factors That Are Associated with Parental Acceptance of Human Papillomavirus Vaccines: A Randomized Intervention Study of Written Information About HPV. Pediatrics.

[17] Galbraith K.V., Lechuga J., Jenerette C.M., Moore L.A., Palmer M.H., Hamilton J.B. (2016). Parental acceptance and uptake of the HPV vaccine among African-Americans and Latinos in the United States: A literature review. Soc. Sci. Med..

[18] Brewer N.T., Gottlieb S.L., Reiter P.L., McRee A.L., Liddon N., Markowitz L., Smith J.S. (2011). Longitudinal predictors of human papillomavirus vaccine initiation among adolescent girls in a high-risk geographic area. Sex. Transm. Dis..

[19] Fu L.Y., Zimet G.D., Latkin C.A., Joseph J.G. (2019). Social Networks for Human Papillomavirus Vaccine Advice Among African American Parents. J. Adolesc. Health.

[20] East K., McNeill A., Thrasher J.F., Hitchman S.C. (2021). Social norms as a predictor of smoking uptake among youth: A systematic review, meta-analysis and meta-regression of prospective cohort studies. Addiction.

[21] Kana’iaupuni S.M., Donato K.M., Thompson-Colón T., Stainback M. (2005). Counting on Kin: Social Networks, Social Support, and Child Health Status*. Soc. Forces.

[22] Staras S.A., Vadaparampil S.T., Livingston M.D., Thompson L.A., Sanders A.H., Shenkman E.A. (2015). Increasing Human Papillomavirus Vaccine Initiation Among Publicly Insured Florida Adolescents. J. Adolesc. Health.

[23] U.S. Department of Health and Human Services (DHHS), National Center for Immunization and Respiratory Diseases The 2019 National Immuization Survey-Teen. https://www.cdc.gov/nis/media/pdfs/2024/11/NIS-TEEN-PUF19-DUG.pdf.

[24] Zimet G.D., Powell S.S., Farley G.K., Werkman S., Berkoff K.A. (1990). Psychometric characteristics of the Multidimensional Scale of Perceived Social Support. J. Pers. Assess..

[25] Valente T.W. (2010). Social Networks and Health: Models, Methods, and Applications.

[26] Liddon N.C., Hood J.E., Leichliter J.S. (2012). Intent to receive HPV vaccine and reasons for not vaccinating among unvaccinated adolescent and young women: Findings from the 2006–2008 National Survey of Family Growth. Vaccine.

[27] Krieger J.L., Sarge M.A. (2013). A Serial Mediation Model of Message Framing on Intentions to Receive the Human Papillomavirus (HPV) Vaccine: Revisiting the Role of Threat and Efficacy Perceptions. Health Commun..

[28] Valente T.W. (2010). Appendix B: Sample Sociometric Survey. Social Networks and Health: Models, Methods, and Applications.

[29] Pedersen E.R., Miles J.N.V., Ewing B.A., Shih R.A., Tucker J.S., D’Amico E.J. (2013). A longitudinal examination of alcohol, marijuana, and cigarette perceived norms among middle school adolescents. Drug Alcohol. Depend..

[30] Shelley G.A., Killworth P.D., Bernard H.R., McCarty C., Johnsen E.C., Rice R.E. (2006). Who knows your HIV status II? Information propagation within social networks of seropositive people. Hum. Organ..

[31] Perry B.L., Pescosolido B.A., Borgatti S.P. (2018). Sociocentric and Egocentric Approaches to Networks. Egocentric Network Analysis.

[32] Shulman J.L., Gotta G., Green R.-J. (2012). Will Marriage Matter? Effects of Marriage Anticipated by Same-Sex Couples. J. Fam. Issues.

[33] Hallgren K.A., Barnett N.P. (2016). Briefer assessment of social network drinking: A test of the Important People Instrument-5 (IP-5). Psychol. Addict. Behav..

[34] Varda D., Retrum J., Kuenzi K. (2012). The Influence of Teaching Methodology on Student Social Interaction. J. Public Aff. Educ..

[35] Dailey R.M., McCracken A.A., Romo L.K. (2011). Confirmation and Weight Management: Predicting Effective Levels of Acceptance and Challenge in Weight Management Messages. Commun. Monogr..

[36] Ngozi Dike S., Freysteinson M.W. (2021). Factors Associated with African American Mothers’ Perceptions of Human Papillomavirus Vaccination of Their Daughters: An Integrated Literature Review. Oncol. Nurs. Forum.

[37] Cunningham-Erves J., Forbes L., Ivankova N., Mayo-Gamble T., Kelly-Taylor K., Deakings J. (2018). Black mother’s intention to vaccinate daughters against HPV: A mixed methods approach to identify opportunities for targeted communication. Gynecol. Oncol..

[38] Valente T.W., Vega Yon G.G. (2020). Diffusion/Contagion Processes on Social Networks. Health Educ. Behav..

[39] Sonawane K., Zhu Y., Montealegre J.R., Lairson D.R., Bauer C., McGee L.U., Giuliano A.R., Deshmukh A.A. (2020). Parental intent to initiate and complete the human papillomavirus vaccine series in the USA: A nationwide, cross-sectional survey. Lancet Public Health.

[40] American Association for Public Opinion Rsearch (AAPOR) Response Rates Calculator. https://aapor.org/response-rates/.

[41] Pietromonaco P.R., Collins N.L. (2017). Interpersonal mechanisms linking close relationships to health. Am. Psychol..

[42] Rini C., Jandorf L., Goldsmith R.E., Manne S.L., Harpaz N., Itzkowitz S.H. (2011). Interpersonal influences on patients’ surgical decision making: The role of close others. J. Behav. Med..

[43] Gilmore B., Gerlach N., Abreu Lopes C., Diallo A.A., Bhattacharyya S., de Claro V., Ndejjo R., Nyamupachitu Mago E., Tchetchia A. (2022). Community engagement to support COVID-19 vaccine uptake: A living systematic review protocol. BMJ Open.

[44] Yewell D., Bentley R.A., Horne B.D. (2024). Perceived Social Influence on Vaccination Decisions: A COVID-19 Case Study. arXiv.

[45] Moore S., Kawachi I. (2017). Twenty years of social capital and health research: A glossary. J. Epidemiol. Community Health.

[46] Gruber K.J., Haldeman L.A. (2009). Using the Family to Combat Childhood and Adult Obesity. Prev. Chronic Dis..

[47] Birditt K.S., Tighe L.A., Fingerman K.L., Zarit S.H. (2012). Intergenerational relationship quality across three generations. J. Gerontol. B Psychol. Sci. Soc. Sci..

[48] Rice E., Klein W. (2019). Interactions Among Perceived Norms and Attitudes About Health-Related Behaviors in U.S. Adolescents. J. Health Psychol..

[49] Berkman L.F., Glass T. (2000). Social Integration, Social Networks, Social Support, and Health. Soc. Epidemiol..

[50] Galbraith-Gyan K.V., Lechuga J., Jenerette C.M., Palmer M.H., Moore A.D., Hamilton J.B. (2019). HPV vaccine acceptance among African-American mothers and their daughters: An inquiry grounded in culture. Ethn. Health.

[51] Haber M.G., Cohen J.L., Lucas T., Baltes B.B. (2007). The relationship between self-reported received and perceived social support: A meta-analytic review. Am. J. Community Psychol..

[52] Ross L., Greene D., House P. (1977). The “false consensus effect”: An egocentric bias in social perception and attribution processes. J. Exp. Soc. Psychol..

[53] Bruine de Bruin W., Parker A.M., Galesic M., Vardavas R. (2019). Reports of social circles’ and own vaccination behavior: A national longitudinal survey. Health Psychol..

[54] Graupensperger S., Abdallah D.A., Lee C.M. (2021). Social norms and vaccine uptake: College students’ COVID vaccination intentions, attitudes, and estimated peer norms and comparisons with influenza vaccine. Vaccine.

[55] Florida Department of Health Population Atlas Dashboard. https://www.flhealthcharts.gov/ChartsReports/rdPage.aspx?rdReport=PopAtlas.PopulationAtlasDASHBOARD&rdRequestForwarding=Form.

[56] National Academies of Sciences, Engineering, and Medicine, Policy and Global Affairs, Committee on Women in Science, Engineering, and Medicine, Committee on Policies and Practices for Supporting Caregivers Working in Science, Engineering, and Medicine (2024). Supporting Family Caregivers in STEMM: A Call to Action.

[57] Argyris Y.A., Kim Y., Roscizewski A., Song W. (2021). The mediating role of vaccine hesitancy between maternal engagement with anti- and pro-vaccine social media posts and adolescent HPV-vaccine uptake rates in the US: The perspective of loss aversion in emotion-laden decision circumstances. Soc. Sci. Med..

[58] Thompson E.L., Preston S.M., Francis J.K.R., Rodriguez S.A., Pruitt S.L., Blackwell J.-M., Tiro J.A. (2022). Social Media Perceptions and Internet Verification Skills Associated with Human Papillomavirus Vaccine Decision-Making Among Parents of Children and Adolescents: Cross-sectional Survey. JMIR Pediatr. Parent..

[59] Wright K., Fisher C., Rising C., Burke-Garcia A., Afanaseva D., Cai X. (2019). Partnering with Mommy Bloggers to Disseminate Breast Cancer Risk Information: Social Media Intervention. J. Med. Internet Res..

