# Supplementary material for: The Role of Personal Social Networks in Parental Decision-Making for HPV Vaccination: Examining Support and Norms Among Florida Parents
Source: Vaccines (Basel). 2025 Jun 21;13(7):667. doi: 10.3390/vaccines13070667 (PMC12300067; doi:10.3390/vaccines13070667)
Supplement: Supplementary file 1 [file vaccines-13-00667-s001.zip › vaccines-3649787-supplementary.pdf]

Supplementary Material

Survey questions are provided verbatim in the order they appear in the manuscript tables. Anywhere [child_name] appears, REDCap filled in the child's first name.

Intention to Vaccinate

11. How likely is it that your child will receive the HPV shots in the next 12 months?

- ◯ Very likely
- ◯ Somewhat likely
- ◯ Not too likely
- ◯ Not at all likely
- ◯ Don't know

Relationship to child

What is your relationship to [child_name]?

- ◯ Biological or Adoptive Parent
- ◯ Step-Parent
- ◯ Grandparent
- ◯ Foster Parent
- ◯ Aunt or Uncle
- ◯ Other

Please specify ______________________________

Gender of respondent

What is your gender?

- ◯ Male
- ◯ Female
- ◯ Other

Other, please specify: ______________________________

Race/Ethnicity of respondent

Are you of Hispanic or Latino origin?

- ◯ Yes
- ◯ No

Please choose one or more of the following categories to describe your race.

- ☐ White
- ☐ Black or African American
- ☐ Asian
- ☐ American Indian or Alaska Native Native
- ☐ Hawaiian or Other Pacific Islander

Age of respondent

What is your current age? Please enter a number ______________________________

Number of 11- to 12-year-old boys and girls of respondent

How many 11- to 12-year-old children are you the caretaker for? (please enter a number): Girl(s)	________________________
How many 11- to 12-year-old children are you the caretaker for? (please enter a number): Boy(s)	________________________

Education level of respondent

What is the highest grade or year of school you have completed?

- ◯ 8th grade or less
- ◯ 9th-12th grade, no diploma
- ◯ High school graduate or GED
- ◯ Vocational, trade, or business school program
- ◯ Some college credit, no degree
- ◯ Associate's degree
- ◯ Bachelor's degree
- ◯ Master's degree (e.g., MBA, MA)
- ◯ Doctorate (PhD, EdD) or professional degree (MD, DDS, DVM, JD)

Marital status of respondent

What is your marital status?

- ◯ Never Married
- ◯ Not married, but living together
- ◯ Married
- ◯ Separated
- ◯ Divorced
- ◯ Widowed

Reporting confidants' questions

Think about the first person you would talk to about important confidential matters and answer the following questions 18 through 22 about this person.

18. How do you know this person?

- ◯ Family member
- ◯ Friend
- ◯ Neighbor
- ◯ Work colleague
- ◯ Other ___
- ◯ I talk to no one about important confidential matters

Other, please specify ________________________

19. How supportive of your decisions is this person?

- ◯ Extremely Supportive
- ◯ Very Supportive
- ◯ Somewhat Supportive
- ◯ Not at All Supportive

20. How influential is this person in your life?

- ◯ Extremely Influential
- ◯ Very Influential
- ◯ Somewhat Influential
- ◯ Not at All Influential

21. How likely is this person to suggest the HPV vaccine for your child?	◯ Very likely<br>◯ Somewhat likely<br>◯ Not too likely<br>◯ Not at all likely
22. What hesitations would this person have about your child getting the HPV vaccine (select all that apply)?	☐ None<br>☐ Vaccine safety<br>☐ Receiving the vaccine at ages 11-to 12-years-old<br>☐ Sexual activity<br>☐ Cost<br>☐ Other ___<br>☐ I don't know/Unsure
Other, please specify	________________________________

Think about the second person you would talk to about important confidential matters and answer questions 23 through 27 about this person.

23. How do you know this person?	◯ Family member<br>◯ Friend<br>◯ Neighbor<br>◯ Work colleague<br>◯ Other ___<br>◯ I cannot think of a second person
Other, please specify	________________________________
24. How supportive of your decisions is this person?	◯ Extremely Supportive<br>◯ Very Supportive<br>◯ Somewhat Supportive<br>◯ Not at All Supportive
25. How influential is this person in your life?	◯ Extremely Influential<br>◯ Very Influential<br>◯ Somewhat Influential<br>◯ Not at All Influential
26. How likely is this person to suggest the HPV vaccine for your child?	◯ Very likely<br>◯ Somewhat likely<br>◯ Not too likely<br>◯ Not at all likely
27. What hesitations would this person have about your child getting the HPV vaccine (select all that apply)?	☐ None<br>☐ Vaccine safety<br>☐ Receiving the vaccine at ages 11-to 12-years-old<br>☐ Sexual activity<br>☐ Cost<br>☐ Other ___<br>☐ I don't know/Unsure
Other, please specify	________________________________

Think about the third person you would talk to about important confidential matters and answer questions 28 through 32 about this person.

28. How do you know this person?	◯ Family member<br>◯ Friend<br>◯ Neighbor<br>◯ Work colleague<br>◯ Other ___<br>◯ I cannot think of a third person
Other, please specify	______________________
29. How supportive of your decisions is this person?	◯ Extremely Supportive<br>◯ Very Supportive<br>◯ Somewhat Supportive<br>◯ Not at All Supportive
30. How influential is this person in your life?	◯ Extremely Influential<br>◯ Very Influential<br>◯ Somewhat Influential<br>◯ Not at All Influential
31. How likely is this person to suggest the HPV vaccine for your child?	◯ Very likely<br>◯ Somewhat likely<br>◯ Not too likely<br>◯ Not at all likely
32. What hesitations would this person have about your child getting the HPV vaccine (select all that apply)?	☐ None<br>☐ Vaccine safety<br>☐ Receiving the vaccine at ages 11-to 12-years-old<br>☐ Sexual activity<br>☐ Cost<br>☐ Other ___<br>☐ I don't know/Unsure
Other, please specify	______________________

Perceived norms from family and friends

14. My family would want me to get the HPV vaccine for my child.

◯ Definitely yes
◯ Probably yes
◯ Possibly
◯ Probably not
◯ Not at all

15. My close friends would want me to get the HPV vaccine for my child.

◯ Definitely yes
◯ Probably yes
◯ Possibly
◯ Probably not
◯ Not at all

Perceived norms to vaccinate

16. Think about a group of 100 parents who have a son of your child's age. How many of them do you think have gotten the HPV vaccine for their son?

- ○ 0-9
- ○ 10-19
- ○ 20-29
- ○ 30-39
- ○ 40-49
- ○ 50-59
- ○ 60-69
- ○ 70-79
- ○ 80-89
- ○ 90-99
- ○ 100

17. Think about a group of 100 parents who have a daughter of your child's age. How many of them do you think have gotten the HPV vaccine for their daughter?

- ○ 0-9
- ○ 10-19
- ○ 20-29
- ○ 30-39
- ○ 40-49
- ○ 50-59
- ○ 60-69
- ○ 70-79
- ○ 80-89
- ○ 90-99
- ○ 100
